# KIT-5 Structural and Textural Changes in Response to Different Methods of Functionalization with Sulfonic Groups

**DOI:** 10.3390/ijms24032165

**Published:** 2023-01-21

**Authors:** Sylwia Chałupniczak, Izabela Nowak, Agata Wawrzyńczak

**Affiliations:** Faculty of Chemistry, Adam Mickiewicz University in Poznań, Uniwersytetu Poznańskiego 8, 61-614 Poznań, Poland

**Keywords:** functionalized KIT-5 mesoporous silica, co-condensation, grafting, structural/textural parameters, solid acid catalyst

## Abstract

In this project, KIT-5 materials were effectively functionalized with sulfonic groups introduced by grafting or the co-condensation method and tested as heterogeneous solid acid catalyst. A co-condensation procedure leading to the stable, –SO_3_H functionalized KIT-5 materials was successfully established. Moreover, the influence of both synthesis methods on the structural and textural parameters, as well as surface chemistry, morphology, and catalytic activity of –SO_3_H/KIT-5 materials was thoroughly investigated. The syntheses with 3-mepkaptopropyltrimethoxysilane (MPTMS) acting as a modifying agent resulted in samples in which functional groups were introduced into the structure and/or onto the mesoporous silica surface. The oxidation stage of –SH to –SO_3_H groups was carried out under mild conditions, using a “green” oxidant (H_2_O_2_). The application of different functionalization techniques and the introduction of different amounts of modifying agent allowed for an evaluation of the influence of these parameters on the ordering of the mesoporous structure of KIT-5 materials. The applied methods of assessment of the physicochemical parameters (XRD, low-temperature N_2_ sorption, TEM) showed that, especially when the co-condensation method was applied, as the number of functional groups increased, the ordering of structure characteristic of KIT-5 decreased. On the other hand, the samples modified by grafting had a stable structure, regardless of the amount of introduced MPTMS. Test reactions carried out on the basis of Friedel–Crafts alkylation process showed that the synthesized materials can be considered promising acid catalysts in heterogeneous catalysis reactions.

## 1. Introduction

Nanostructured materials have been of continuing interest to researchers in recent years due to their unique properties and a wide range of potential applications in various areas [1,2,3,4]. Particular attention has been paid to densely packed structures of constant periodicity and high order, including mesoporous silica materials, which have large specific surface areas, high thermal and mechanical stability, well defined pore systems, easily adjustable physical and chemical properties, and can be functionalized. Moreover, the cost of their production is relatively low and the synthesis procedure is quite simple [5,6,7,8].

Due to the above-mentioned properties, ordered mesoporous silica materials have become very good carriers for heterogeneous solid catalysts. Heterogenic nanocatalysts have a number of advantages, such as stability, easy separation from the reaction environment and possibility of reuse [9,10,11]. Solid catalysts containing acidic centers are an ecologically important alternative to homogeneous catalysts employed to obtain the high quality chemicals used in various industries, e.g., petrochemical, pharmaceutical, and cosmetic [12,13,14,15].

**Figure 1:**
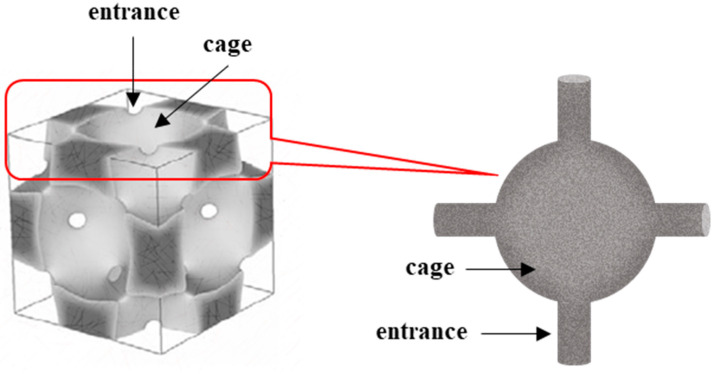
Structure of KIT-5 material (**left side**) and scheme of pore system characteristics of this type of material (**right side**).

Among acidic solid catalysts, mesoporous silica materials modified with sulfonic groups present an interesting option, as due to their highly developed specific surface area they can provide a large number of available active centers and, thanks to their defined pore size, they can support the reaction selectivity [10,16,17,18,19]. It seems interesting to use for this purpose KIT-5 materials, which were first synthesized by Ryoo research group [20] in 2003. The KIT-5 material is attractive due to its three-dimensional, very well structured, face-centered cage-type cubic structure of Fm3m symmetry (Figure 1) [21,22]. In this structure, each nano-sized cage is connected to 12 adjacent nanocages by means of entrances (inlets) of significantly smaller sizes [23,24]. Such a three-dimensional system of interconnected pores is considered more advantageous than the other systems in different mesoporous silica materials as it leads to faster diffusion of the reagents to the active centers, prevents their blocking, and can be used to transform larger-size particles [25,26,27].

Despite such interesting structural features, unmodified KIT-5 materials are characterized by poor surface acidity and poor ion exchange capacity, which limits the potential of their applications. These problems can be overcome by functionalization of these materials with the appropriate functional groups [24,28,29,30,31,32].

The most commonly used compound, which allows for the introduction of sulfonic groups into the structure and/or onto the surface of mesoporous silica, are organosilanes, i.e., 3-mercaptopropyltrimethoxysilane (MPTMS). The molecule of this compound contains a thiol group –SH, a stable propyl group and a hydrolysable residue –Si(OCH_3_)_3_. This allows MPTMS to be introduced by both grafting (post synthesis) and co-condensation (one-pot). The key step to obtaining sulfonic group modified materials using this precursor is the oxidation of thiol to sulfonic groups. The application of H_2_O_2_ as a “green” oxidant permits running this process in mild conditions, often at room temperature [10].

Methods for silica materials functionalization by grafting and co-condensation have already been described in the literature (e.g., Refs. [33,34]). However, we do not find numerous reports on the effect of the applied technique of sulfonic groups introduction on the preservation of the ordered three-dimensional mesoporous structure in KIT-5 materials. Therefore, the aim of the presented studies was to develop a simple, effective, and environmentally friendly method for the synthesis of ordered nanostructured silicas of KIT-5 type modified with different amounts of sulfonic groups, as well as to determine the effect of the synthesis parameters on the physicochemical properties and catalytic activity of the materials obtained. Two different methods of introducing functional groups into the silica matrix were used: grafting, which involves modification of previously synthesized silica materials, and co-condensation, where the formation of the silica matrix takes place in the presence of the modifying agent. The physicochemical properties of the obtained materials were evaluated using various techniques, namely: X-ray diffraction (XRD) in the low angle range 2θ, low temperature nitrogen adsorption/desorption, transmission electron microscopy (TEM), and elemental analysis. The concentration of acidic active centers was checked using acid-base titration in the presence of phenolphthalein as an indicator. The catalytic activity of the synthesized KIT-5 materials was tested in the Friedel–Crafts alkylation reaction, which is considered one of the most useful reactions of carbon-carbon bond formation in aromatic systems [35,36]. In this project, KIT-5 was effectively functionalized with sulfonic groups by grafting and co-condensation method and used as a heterogeneous solid acid catalyst. A co-condensation procedure leading to stable, –SO_3_H functionalized KIT-5 materials was successfully established. Moreover, the influence of both synthesis methods on the structural and textural parameters, as well as surface chemistry, morphology, and catalytic activity of –SO_3_H/KIT-5 materials was thoroughly investigated.

## 2. Results

### 2.1. Characterization of KIT-5 Based Materials

#### 2.1.1. X-ray Diffraction (XRD)

**Figure 2:**
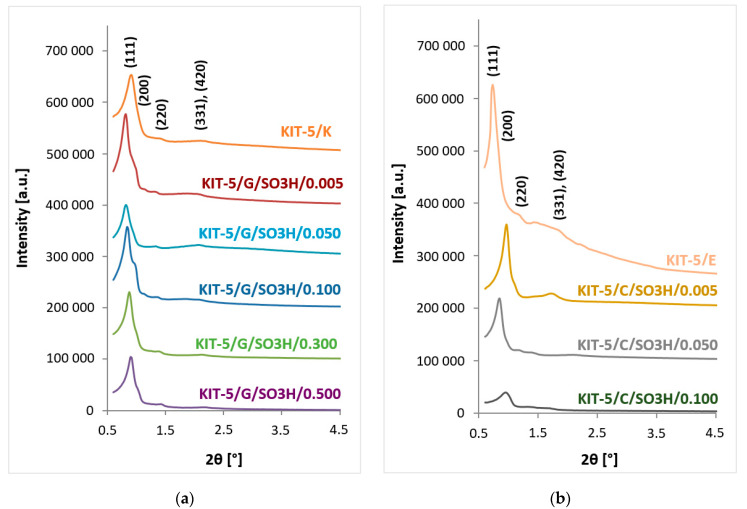
Diffractograms of KIT-5 type materials modified with sulfonic groups by (**a**) grafting or (**b**) co-condensation.

The XRD studies were carried out in order to obtain information on the structure of the material and the degree of its ordering. The diffractograms of low angle 2θ obtained for KIT-5 type materials modified with an increasing number of sulfonic groups introduced by grafting or co-condensation are depicted in Figure 2. The diffractograms in Figure 2a show reflections at Bragg’s angle of about 0.8°, 1.0°, 1.4°, and 2.2°, corresponding to the lattice planes (111), (200), (220), and (331) and (420), which are characteristic of materials with a well-ordered structure of symmetry Fm3m [20,37]. This proves that the synthesized materials are characterized by a very well-ordered and stable structure of mesopores. On the other hand, the diffractograms presented in Figure 2b clearly indicate that as the number of functional groups introduced by co-condensation increases, the XRD pattern characteristic of KIT-5 changes, namely the breadth of the main peak (111) increases and its maximum shifts towards higher 2θ values, specifying that the degree of ordering of the obtained materials is reduced. The greatest decrease in the degree of the structure ordering was visible for the sample KIT-5/C/SO_3_H/0.100, therefore KIT-5 materials with the molar ratio TEOS:MPTMS greater than 1:0.100 were left out of the study. The mesoporous structure with the highest degree of ordering is characterized by the purely siliceous material (KIT-5/E); however, on the diffractogram of the sample containing a smaller number of functional groups introduced by co-condensation there also appear distinct reflections characteristic of materials with cubic structure and Fm3m symmetry, which KIT-5 type silica materials have. It can also be noted that in the diffractogram for the KIT-5/E material, the position of the main peak (111) is shifted towards lower 2θ values than in the case of the modified materials. This will also be reflected in the values of the parameters d_111_ and a_0_ (Table 1).

The samples containing sulfonic groups introduced by grafting are characterized by similar values of structural parameters (Table 1), i.e., distances between planes (111) and unit cell sizes. In addition, they are close to the values obtained for the initial KIT-5/K sample, which confirms the negligible effect of such modifications on the structure of the vehicle subjected to the grafting process. However, for the samples obtained by co-condensation, it is clear that as the amount of introduced MPTMS increases, the structural parameters change. This confirms a much greater influence of modifications induced by co-condensation. These changes become much more apparent when samples modified by co-condensation are compared with the KIT-5/E reference material subjected to an extraction process to remove the structure-directing agent, than is the case with samples modified by grafting and compared with the KIT-5/K starting material, from which the template was removed by calcination.

Co-condensation involves the addition of a modifying agent (in our case MPTMS) at the stage of mesoporous silica matrix formation. This has a significant impact on the parameters of the forming mesoporous structure. Hydrolyzed MPTMS molecules may interact with the block co-polymer (Pluronic F127). This interaction may cause changes in the micelles that form the mesophase. This change might not be compatible with the KIT-5 structure, thus, turning the mesophase unstable and creating fluctuations in the cell parameters. According to the previous studies, adding the modifying agent during the synthesis may cause a distinct segregation of silica matrix and large ethoxy domains stemming from F127 copolymer in the mesophase [38,39]. Moreover, per analogue to Vegard’s law [40], we may assume that the first “portions” of sulfonic groups in the reaction mixture are quite homogeneously distributed and rather order the matrix—that is why the lattice parameter (a_0_) decreases and is lower than that of the unmodified counterpart (KIT-5/E) and other samples modified by co-condensation. With the increasing dopant concentrations, the distribution of the sulfonic groups precursor in the matrix becomes more irregular (inhomogeneous). Thus, the stress in the forming siliceous matrix will significantly affect the unit cell parameters, which is the most evidenced for the sample KIT-5/C/SO_3_H/0.100, especially when compared with an unmodified counterpart, namely KIT-5/E (Table 1).

It should be kept in mind that, in the case of samples modified by co-condensation, a separate synthesis is necessary since functional groups cannot be introduced by this method into the already finished silica material. Therefore, significant differences may be observed in terms of the change in the values of the parameters d_111_ and a_0_ obtained for samples modified with sulfonic groups introduced by grafting and co-condensation relative to their initial counterparts, namely KIT-5/K and KIT-5/E. For the samples involved in the grafting procedure, namely KIT-5/G/SO_3_H/0.005 and KIT-5/K, the parameters d_111_ and a_0_ were 10.8 nm and 18.8 nm, respectively, for the modified material and 10.5 nm and 18.3 nm, respectively, for the pure silica sample, while for the co-condensed material KIT-5/C/SO_3_H/0.005, the parameters d_111_ and a_0_ were 9.1 nm and 15.7 nm, and for the starting material KIT-5/E they were 12.0 nm and 20.8 nm, respectively. It can be assumed that such large differences are due to the way the latter materials were acquired: the KIT-5/C/SO_3_H/0.005 and KIT-5/E samples were obtained by two separate synthesis processes, and in addition, they were subjected to the extraction for removing the structure-directing agent, which does not involve such a strong shrinkage of the silica structure resulting from condensation of silanol groups, as is the case with calcination. In addition, it may leave some of the template not removed from the porous structure, especially from smaller pores. Hence, such pronounced differences can be observed in structural parameters between these two reference samples, namely KIT-5/K and KIT-5/E, despite the fact that they were obtained in the same synthesis cycle and the only difference was the template removal method.

#### 2.1.2. Low-Temperature Nitrogen Sorption

**Figure 3:**
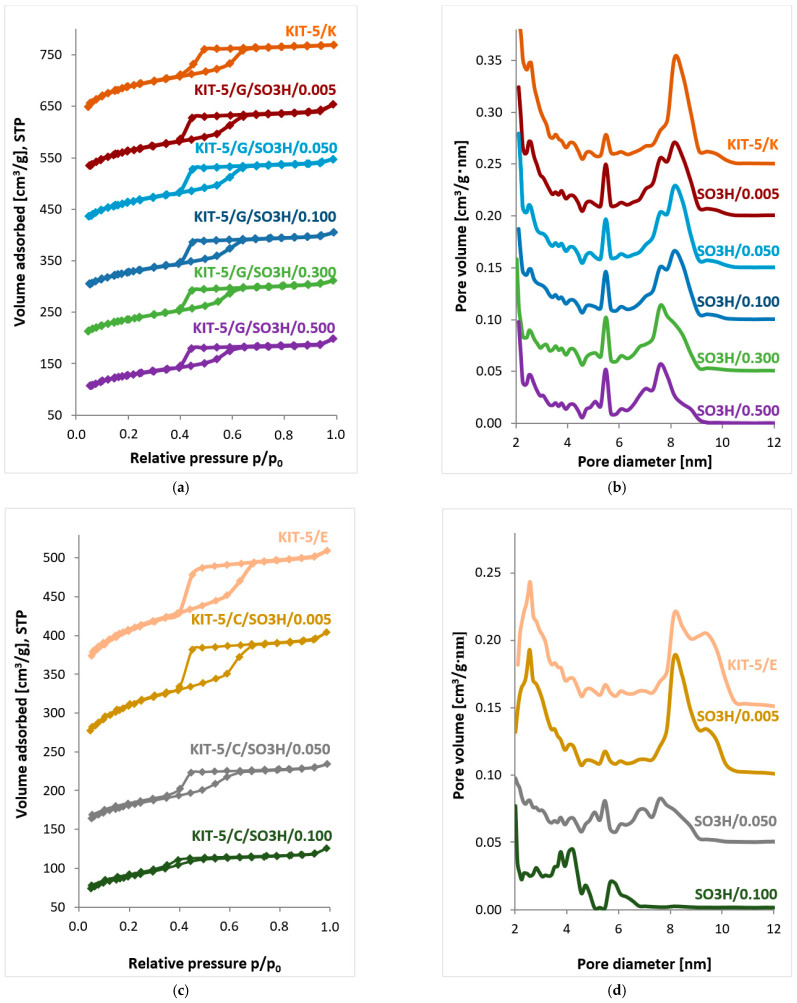
Low-temperature nitrogen sorption isotherms and pore size distributions (N_2_ at 77 K on the silica–cylindrical/spherical pore, NLDFT ads. Model) for KIT-5 type materials modified with sulfonic groups by (**a**,**b**) grafting or (**c**,**d**) co-condensation. The isotherms were shifted by 100 cm^3^/g STP and the pore size distribution curves by 0.05 cm^3^/g·nm.

Low-temperature nitrogen sorption isotherms for KIT-5 type materials modified with an increasing amounts of MPTMS are shown in Figure 3a,c. According to the IUPAC specifications, most of the depicted isotherms have shapes typical for mesoporous materials [41]. The sizes and shapes of hysteresis loops for the samples obtained by grafting are almost identical, which indicates similar amounts of absorbed nitrogen and similar mechanisms of the desorption process. On the other hand, the isotherms obtained for non-modified sample KIT-5/E and the materials modified by co-condensation at the molar ratio TEOS:MPTMS of 1:0.005 or 1:0.050, show well-formed hysteresis loops in the relative pressure range p/p_0_ 0.40–0.64. In contrast, the isotherm recorded for the material containing the largest number of functional groups introduced by co-condensation, i.e., KIT-5/C/SO_3_H/0.100, shows negligible hysteresis loop. Thus, the proportion of mesopores in the samples synthesized by co-condensation decreases gradually as the number of introduced functional groups increases. This finding confirms a significant effect of co-condensation, already observed in XRD measurement results, on the ordering of the structure of the obtained materials. The pore size distributions obtained for the samples grafted with MPTMS (Figure 3b) clearly indicate a wide range of pore sizes, i.e., from 2 nm to 10 nm. For the samples to which from 0.005 to 0.100 moles of MPTMS were introduced in relation to 1 mole of TEOS, the maximum cage diameters are close to 8.2 nm, while the maximum cage inlet diameters are of about 2.6 nm. With the increasing number of introduced functional groups, the cage diameters decrease, which is proved by the maximum cage size of about 7.6 nm and the increase in the cage inlet diameters to 2.7 nm. For all such samples, the maximum pore diameter between the adjacent cages is 5.5 nm. Nonetheless, it should be noticed that the grafting process is prone to diffusional limitations [7,42]. Thus, the organosilane may not reach the very bulk of the particles; the consequence of which is the functionalization of pores situated at more accessible locations. This will create a shoulder (at the left side) in the peak from the primary pore size, as observed in Figure 3b. However, as expected, the distribution of pore sizes for samples obtained by co-condensation (Figure 3d) is much different. The pore size distribution for the KIT-5/E material shows the presence of two maxima for cage pores: one at 8.2 nm and the other at 9.4 nm. Furthermore, it is clear from the nitrogen sorption curves obtained that as the number of introduced functional groups increases, the pore diameters and volumes decrease. While the maximum cage diameter for KIT-5/C/SO_3_H/0.005 is 8.2 nm and the size of the cage entrances is 2.6 nm, the pore size distribution for KIT-5/C/SO_3_H/0.100 already differs significantly from those obtained for the other samples. Moreover, a shoulder at the right side of the peak from primary pores can be observed (Figure 3d). Its presence is most likely caused by non-homogeneous pore expansion during mesophase formation [37]. Instability and the changes of the mesophase, which are not compatible with the KIT-5 structure, are most likely caused by transformations in the micelles that form the mesophase. This, in turn, may be induced by interactions between the organic group present in the MPTMS and the block co-polymer, F127.

The KIT-5 type materials modified with sulfonic groups have good textural parameters (Table 2), although the samples obtained by co-condensation show lower specific surface areas, total pore volume and pore diameter, when compared to the analogous samples obtained by grafting. It was also indicated that the pore wall thicknesses in the samples obtained by co-condensation are greater than those of the samples obtained by grafting, which was confirmed by the fact that the functional groups introduced by co-condensation influence the process of silica matrix formation.

The micropore volume for each sample was calculated on the basis of the alpha-s model. The presence of micropores should be correlated with the sizes of the pore entrances, which take values from the border between micro- and mesopores. Although the exact values of the pore entrances’ diameters cannot be calculated on the basis of low-temperature N_2_ sorption measurements, it seems that the presence of the peak in the region of the pore diameters close to 3.0 nm in the pore size distributions (Figure 3b), might be attributed to the pore entrance of the cage-like mesopores [43]. Furthermore, the points of closing of the desorption branches in the isotherms, also point to the fact the entrances to the mesoporous cavities may be regarded as smaller than 5.0 nm [20].

**Figure 4:**
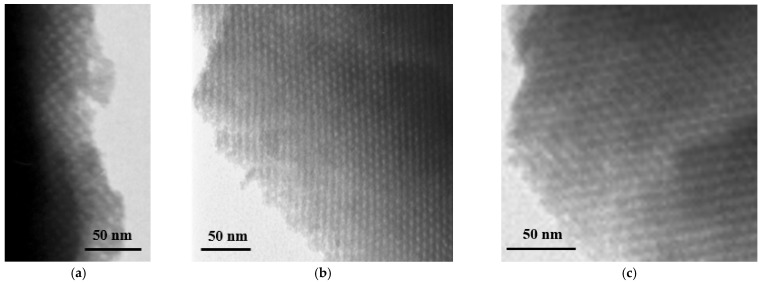

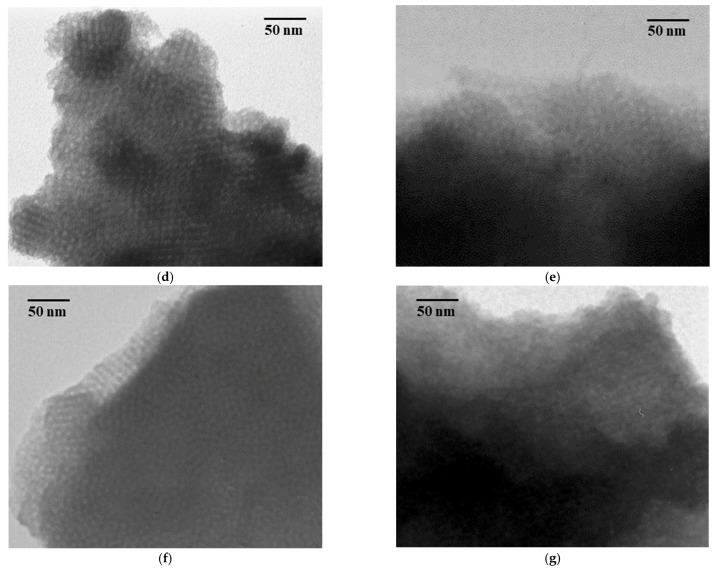
TEM photos of (**a**) KIT-5/K material and samples modified with sulfonic groups by grafting: (**b**) KIT-5/G/SH/0.100, (**c**) KIT-5/G/SO_3_H/0.100, (**d**) KIT-5/E material, and samples modified by co-condensation: (**e**) KIT-5/C/SO_3_H/0.005, (**f**) KIT-5/C/SO_3_H/0.050, (**g**) KIT-5/C/SO_3_H/0.100.

As it was mentioned before, co-condensation involves the addition of a modifying agent at the stage of formation of the mesoporous silica matrix. This has a significant effect on the parameters in the forming mesoporous structure. Thus, the structural and textural parameters may vary upon the amount of the added modifying agent. It may be observed in Figure 3c, where all the displayed isotherms are of type IV(a), whereas the hysteresis loops gradually lose their H2 shape, attributed to the cage-like mesopores. This less distinct capillary condensation step demonstrates smaller mesopore volume [20]. Such a transformation in the feature of the hysteresis loops may reveal the variation in the pore topology, which may also be observed on the plot of the pore size distribution, as well as in the calculated values of D_me_ in Table 2. Change in the matrix structure, when compared with an unmodified counterpart (KIT-5/E), is visible even for the sample functionalized with the smallest amount of MPTMS. Still, somewhat surprising is the large value of D_me_ for the KIT-5/E sample, especially when compared to the KIT-5/C/SO_3_H/0.005 material. This is due to the fact that the calculation of this parameter requires a unit cell parameter (a_0_), which also took a large value for the unmodified sample. In a way, such an increase in cage size may be indicated by the pore size distribution itself (Figure 3d), which shows a higher proportion of pores with diameters >8.2 nm than was the case even for the sample modified with the smallest amount of MPTMS. This may be caused by non-homogeneous pore expansion during creation of the mesophase [37]. This indicates a less uniform but still ordered structure with Fm3m symmetry, as shown by XRD measurements. A similar relationship was also observed by Yang and co-workers who obtained aminopropyl-functionalized mesoporous KIT-5 materials and also found that the non-modified sample has a larger unit cell and cage diameter than is observed for functionalized samples [44]. However, these changes are especially pronounced in the KIT-5/C/SO_3_H/0.100 sample, where the decrease in D_me_ value is accompanied with a significant increase in the value of the wall thickness (up to 10.1 nm). This change, namely a transformation from the cage-like structure to the more cylindrical-based one, leads to the porous material with a still pronounced surface area (308 m^2^/g). Disruption in the 3D cage-like structure can be observed as well in the TEM photos: the porous structure is still visible, though in the less ordered mode (Figure 4g).

#### 2.1.3. Transmission Electron Microscopy (TEM)

The presented series of TEM images (Figure 4a–c) for the KIT-5 type materials modified with sulfonic groups by grafting show that at each stage of synthesis the obtained samples are characterized by an ordered structure with a cubic mesoporous arrangement characteristic of KIT-5 type materials. The introduced amount of MPTMS and the performed process of oxidation of thiol groups to sulfonic ones did not affect the ordering of the structure. This is an additional confirmation of the earlier mentioned observation that the designed procedure of synthesis of KIT-5 materials functionalized by grafting does not affect the degree of ordering of the mesoporous silica structure.

However, when the co-condensation method was used for KIT-5 functionalization, changes in the structure were more noticeable (Figure 4d–g). It can be seen that as the number of introduced functional groups increases, the ordering of the structure slightly decreases. It is noticeable especially for the sample with the highest loading with –SO_3_H groups (Figure 4g), where the regions of the well-ordered and disordered mesoporous structure can be distinguished. Similar conclusions can also be drawn from XRD patterns (Figure 2b).

#### 2.1.4. Elemental Analysis

The results of elemental analysis (Table 3) confirmed the effectiveness of the proposed procedures for the introduction of thiol groups from MPTMS into all synthesized silica samples. As can be seen, the percentage content of sulphur and carbon decreases after the oxidation process, which may indicate leaching of some of the introduced groups. For the samples KIT-5/G/SO_3_H/0.005 and KIT-5/C/SO_3_H/0.005, in which the initial number of functional groups was the smallest, a slight decrease in the number of functional groups is observed after the oxidation process. As expected, the amount of sulphur leaching is higher for the samples subjected to the grafting procedure. This shows a stronger binding of the functional groups introduced by co-condensation in the silica matrix. The modification process efficiency index (R) decreases with the increasing number of functional groups introduced. Comparing the two different modification methods, it can be seen that for the samples containing more MPTMS, the effectiveness of the introduction of functional groups is higher by co-condensation than by grafting. The presented data also indicate that the samples modified by co-condensation contain more carbon and hydrogen than those modified by grafting. The source of these elements may be the surfactant Pluronic F127. Their increased amount is the evidence of a slightly lower efficiency of extraction used instead of traditional calcination to remove the directing agent from the materials synthesised by co-condensation.

#### 2.1.5. Surface Acidity Measurements

Acidity testing of the synthesized catalysts using the acid-base titration technique made it possible to calculate the amount of millimoles of H^+^ ions per 1 g of sample. The results of the analyses carried out by this method, both in the direct and reverse titration options, are presented in Table 4. As expected, the concentration of acidic active centres in the synthesized materials increases with the number of introduced organic functional groups. Additionally, the conversion of the number of active centres per unit area shows a gradual and systematic increase in their number. Comparing the acidity of the sample KIT-5/G/SO_3_H/0.005 with the acidity of the starting material before the functionalization process, i.e., KIT-5/K, it can be concluded that even a small amount of modifying compound increases the acidity (from 0.04 mmol H^+^/g to 0.05 mmol H^+^/g). Moreover, the acidity of the samples modified by co-condensation was much higher than that of those functionalized by grafting. It could be observed even in the sample with the lowest amount of MPTMS, where acidity changes from 0.04 mmol H^+^/g in the pristine KIT-5 material (KIT-5/E) to 0.10 mmol H^+^/g in the KIT-5/C/SO_3_H/0.005 sample. However, it should be noted that despite the fact that the results of the elemental analysis indicate a comparable content of functional groups for the samples KIT-5/G/SO_3_H/0.005 and KIT-5/C/SO_3_H/0.005 (Table 3), quite surprisingly, the results of the acid-base titration show a significant difference in the acidity of their surfaces, namely 0.05 mmol H^+^/g vs 0.10 mmol H^+^/g for KIT-5/G/SO_3_H/0.005 and KIT-5/C/SO_3_H/0.005, respectively. Nevertheless, in the case of samples with the lowest content of functional groups, the results obtained by titration should be approached with some caution, since at such low values they may be subjected to a slightly higher error because of the limitations of this analytical method.

#### 2.1.6. Measurements of Particle Size Distributions

Particle size distributions, where the volume of particles is displayed as a function of the particle size, were plotted on the basis of the laser diffraction measurements for the KIT-5 materials synthesized by co-condensation and grafting procedures (Figure S1).

A significant contribution of large particles (>10 µm) is visible for the sample with the lowest content of –SO_3_H groups (KIT-5/C/SO_3_H/0.0050) but the number of large particles decreases with the amount of introduced functional groups (Figure S1b).

For KIT-5 materials obtained by grafting, the effect of the amount of introduced functional groups on the particles sizes is much more visible. The plot for the parent material (KIT-5/K) shows the presence of two distinct peaks (Figure S1a), while for materials modified with –SO_3_H groups the peak indicating the presence of larger particles is significantly reduced. At the same time the peak representative for particles with smaller sizes is growing. It could be hypothesized that particles between 10 μm and 100 μm represent agglomerates of smaller particles. The grafting procedure is likely to increase the hydrophilicity of the particles and, consequently minimize the formation of agglomerates.

The presented SEM images are somehow in a good agreement with results of the particle size distribution measured by laser diffraction: the particles of the studied materials do not have regular shapes and uniform sizes. However, the samples modified by co-condensation show a slightly lower tendency to form agglomerates than their counterparts obtained by grafting. The KIT-5/K sample has both small and larger particles with fairly smooth surfaces in sizes ranging from 1 µm to 10 µm clustered into agglomerates (Figure S2a). This is most likely the reason why the particle size distribution plot (Figure S1a) for this sample shows an additional signal in the range of 10–100 µm. The SEM images presented in Figure S2f–j show that the modification by co-condensation did not cause significant changes in the morphology of the samples.

### 2.2. Friedel–Crafts Alkylation Reaction

**Figure 5:**
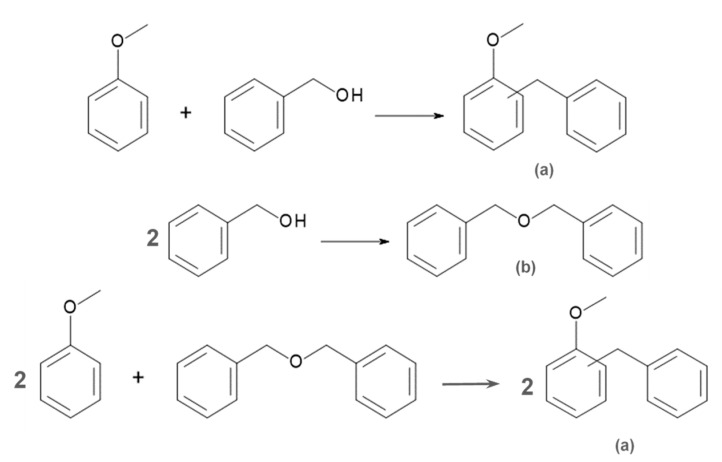
Scheme of the Friedel–Crafts alkylation reaction between anisole and benzyl alcohol, resulting in the following products: (**a**) o-benzylanisole or p-benzylanisole and (**b**) dibenzyl ether.

The catalytic activity of the synthesized nanostructural materials modified with sulfonic groups was tested in the Friedel–Crafts alkylation reaction between anisole and benzyl alcohol. The reactions give three products: o-benzylanisole, p-benzylanisole, and dibenzyl ether (Figure 5), which is an undesirable product formed by the condensation of two molecules of benzyl alcohol.

**Figure 6:**
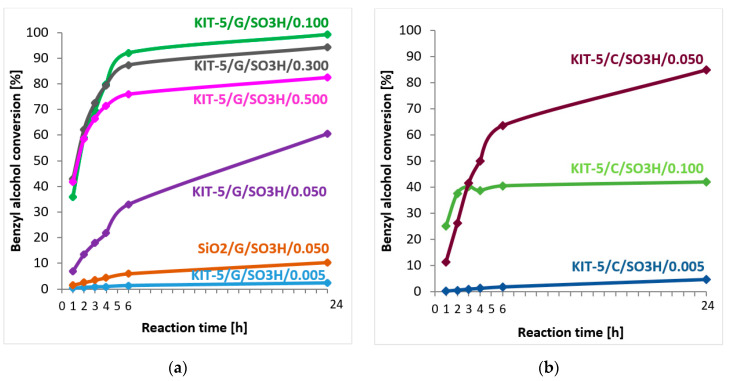
Relationship of benzyl alcohol conversion to the time of Friedel–Crafts alkylation reaction for KIT-5 type materials modified with sulfonic groups by (**a**) grafting or (**b**) co-condensation.

As follows from the results plotted in Figure 6, the materials based on KIT-5 silica modified with sulfonic groups by grafting or co-condensation show catalytic activity in this reaction. However, it can be seen that greater amounts of the introduced functional groups do not cause the expected increase in the conversion of benzyl alcohol. The highest conversion, reaching 100% after 24 h of the reaction, was obtained for KIT-5/G/SO_3_H/0.100, whereas modification with a greater number of functional groups leads to the production of catalysts with surprisingly low activity, which is evidenced by a decrease in benzyl alcohol conversion. For KIT-5/G/SO_3_H/0.500, after 24 h of the reaction, the conversion falls to 82% (Figure 6a). The materials modified by the co-condensation method show a similar trend but the highest value of benzyl alcohol conversion was achieved for the catalyst KIT-5/C/SO_3_H/0.050 (85% after 24 h of the reaction), while the conversion in the presence of KIT-5/C/SO_3_H/0.100 after 24 h was almost twice as low and reached 42% (Figure 6b). The discrepancy between the conversion rates obtained for the samples with the same number of functional groups introduced by co-condensation (KIT-5/C/SO_3_H/0.100) or grafting (KIT-5/G/SO_3_H/0.100) is surprising. Analysis of the degrees of conversion in the presence of the samples obtained by co-condensation considering the structural (Table 1) and textural (Table 2) parameters of this material, implies that the introduced number of functional groups clearly disturbs the ordered mesoporous structure. The functional groups may block part of the pore inlets and hinder the diffusion of substrate molecules in the catalyst channels, which results in a decrease in its catalytic activity in comparison with that of the sample with nominally the same number of functional groups introduced by grafting. In addition, some of the functional groups may have been located in regions of disturbed structures that are not as easily accessible to the reagents as it would have been if a well-structured three-dimensional structure with properly shaped cage pores had been formed. Moreover, functionalization by grafting is more prone to diffusional limitations and thus the molecules of the modifying agent may not reach deeper parts of the material and locate the more accessible fragments of pores. For this reason, the KIT-5/G/SO_3_H/0.100 catalyst may be more catalytically active than its counterpart obtained by co-condensation, in which the functional groups may be located in deeper parts of the pores, becoming in turn less accessible to the reactants’ molecules. The effect of functional group accessibility on the catalytic activity becomes apparent especially for material obtained by co-condensation with the highest amount of modifying agent (KIT-5/C/SO_3_H/0.100). These assumptions are further confirmed by the data obtained from elemental analysis (Table 3), which show that the number of functional groups finally introduced into the catalyst by co-condensation is more than three times higher than in the case of grafting, which should translate into the catalytic activity of this material. However, this is not the case and the sample KIT-5/C/SO_3_H/0.100 is clearly less catalytically active than its equivalent obtained by grafting.

Friedel–Crafts alkylation with commercial silica (SiO_2_) modified with sulfonic groups was carried out analogously to the catalytic tests with KIT-5 materials. Thiol groups were introduced onto the SiO_2_ surface by grafting MPTMS in the amount that corresponded to the volume of this precursor taken during preparation of KIT-5/G/SO_3_H/0.050 sample. Thus, an equivalent of this catalyst was obtained based on commercial silica with disordered arrangement of pores. The results of the catalytic tests show that conversion of benzyl alcohol for SiO2/G/SO_3_H/0.050 catalyst after the 1st hour of the reaction reached only 2%, and after the 6th and 24th hour of the reaction it was 6% and 10%, respectively. The results obtained for the analogous KIT-5/G/SO_3_H/0.050 material show that the catalyst with mesoporous structure is much more active than its counterpart based on the commercial silica, leading to much higher benzyl alcohol conversion (Figure 6). Therefore, it may be concluded that the ordered 3D mesoporous structure of KIT-5 material facilitates reagents distribution in the porous system.

The selectivity values presented in Table 5 for individual products of the reaction catalysed with KIT-5 type nanostructured materials allow us to conclude that the main product is the compound substituted at the para position in relation to the methoxy group present in the anisole molecule, i.e., p-benzylanisole. The second desired product, substituted at the ortho position (o-benzylanisole), is formed in a smaller amount, which results from the effect of the redirecting methoxyl substituent present in the anisole molecule. Dibenzyl ether is formed in the smallest amount. By increasing the reaction time, the amount of both p-benzylanisole and o-benzylanisole increases, while the contribution of dibenzyl ether decreases. Thus, it can be concluded that with the reaction time, some of the resulting dibenzyl ether breaks down and, together with the anisole molecules, takes part in the formation of alkylation products [45]. It is worth noting that when the samples modified by grafting are used, a smaller amount of dibenzyl ether is formed in favour of larger amounts of the desired reaction products. What is more, in the case of KIT-5/G/SO_3_H/0.050 catalyst the amounts of the formed dibenzyl ether are lower than for a catalyst based on the commercial silica. This could most likely be correlated to the shape selectivity of the 3D ordered mesoporous structure of the KIT-5 materials.

In order to check the effect of the mesoporous support on the catalytic activity of the Brönsted acid centers generated by the sulfonyl groups, catalytic tests were also carried out with a homogeneous catalyst, which was p-toluenesulfonic acid (PTSA). The amount of PTSA was chosen so that the number of functional groups involved in the catalytic process was the same as in the test with a KIT-5/C/SO_3_H/0.100 sample, for which the sulphur atom content determined by elemental analysis was the highest (2.74%) among all the synthesized catalysts. The results in Table 5 show that in this particular catalytic system PTSA is characterized by quite poor activity. After 24 h of the reaction, the conversion of benzyl alcohol does not exceed 3%, which is a significantly lower value than that for the analogous heterogeneous catalyst (42%). Furthermore, the distribution of products presents itself differently from that of a heterogeneous system: in the presence of PTSA, the reaction by-product (dibenzyl ether) is mainly formed, and its amount systematically increases with the increasing reaction time. However, the selectivity to the alkylation products shows a principally ortho-para substitution, which is in accordance with a typical electrophilic aromatic substitution pathway. These results clearly indicate the positive influence of the mesoporous silica support. However, it should be noted that the sulfonic groups in the tested catalytic systems have slightly different chemistry—heterogeneous catalysts possess propylsulfonic linkers, while in the p-toluenesulfonic acid molecule the –SO_3_H group is attached to the aromatic group.

## 3. Discussion

In the course of the presented study, it was possible to introduce –SO_3_H groups into the structure of KIT-5 materials, both by co-condensation and grafting, while maintaining the ordered mesoporous structure and keeping the values of structural and textural parameters at an appropriate level. Studies conducted so far on the modification of KIT-5 materials often show a noticeable decrease in the ordering of the structure upon introduction of the modifying agent, especially by co-condensation [6,24,25,26,27]. In the case of our studies it was also observed, but limited mostly to the sample KIT-5/C/SO_3_H/0.100 with the highest amount of the modifying agent introduced by co-condensation.

The XRD technique in the low angle range confirms the stable structure and cubic arrangement of mesopores in the analyzed samples [20]. Analysis of the diffractograms of samples synthesized by grafting shows that grafting an increasing number of functional groups does not result in deterioration of the initial material structure, i.e., that of the material after calcination (KIT-5/K). This proves that these materials are characterized by a very well-ordered and stable structure of mesopores. On the other hand, as the number of functional groups introduced by co-condensation increases, the breadth of the reflections characteristic of KIT-5 decreases, and thus we may assume that the degree of ordering of the obtained materials is reduced.

The samples containing sulfonic groups introduced by grafting are characterized by similar values of structural parameters, which are close to the values obtained for the initial KIT-5/K sample. It confirms the negligible effect of this type of modification on the silica’s ordered mesoporous structure. For the samples obtained by co-condensation, it is clear that as the amount of introduced MPTMS increases, the structural parameters change. It confirms a much greater influence of modifications induced by co-condensation, i.e., during the formation of mesoporous silica matrix, on the sample structure. This is consistent with previously obtained results, e.g., Ref. [24]. It can also be observed that KIT-5 calcined material has a smaller elemental cell size than its counterpart from which the template was removed by extraction, which may be due to shrinkage of the silica matrix due to condensation of the –OH groups present on its surface under the action of high temperature [7].

The shape of N_2_ physisorption isotherms for synthesized KIT-5 samples is consistent with that of type IV(a) isotherms according to IUPAC classification [41], which is characteristic of mesoporous materials. Well-developed hysteresis loops, which occur in the relative pressure range p/p_0_ 0.4–0.6, are assigned to type H2(a) [41], which is characteristic of bottle-shaped pores (narrow inputs, wide interiors), and which are found in KIT-5 type materials. This type of hysteresis loop results from pore blocking and cavitation phenomena upon N_2_ desorption and, as a result, only the adsorption branch is suitable for pore size distribution calculations [46]. Therefore, the NLDFT adsorption model (N_2_ at 77 K on the silica–cylindrical/spherical pore) has been applied during our studies. The obtained pore size distributions confirm that the pore diameters and volumes decrease as the number of introduced functional groups increases, especially for samples modified by the co-condensation route. According to the pore size distributions shown in Figure 3d, samples modified by co-condensation with a TEOS:MPTMS molar ratio of 0.005 possess cage-like pores with diameters between 8 nm and 10 nm and pore entrances between neighboring mesopores of 2 nm and 4 nm. An increase in the amount of introduced functional groups leads to the change of the position of the peak stemming from the sizes of cage-like mesopores; it shifts towards lower values in the case of the KIT-5/C/SO_3_H/0.100 sample. This is accompanied with the reduction of the peak originating from the entrances of cage-like pores, proving that this material possess less widened pore entrances. All these changes in the topology of cage-like mesopores and pore entrances are also reflected by the modifications in the shape of respective hysteresis loops observed in the Figure 3c. Therefore, we have further evidenced that the mesoporous structure of this material was disturbed by the presence of the modifying agent, namely by its interactions with a block co-polymer, which influence the micelles that form the mesophase. It is in accordance with the previous study of Wu et al. who examined structural modulations in KIT-5 mesoporous silica treated by H_2_SO_4_ [37]. Measurements carried out with the use of low-temperature nitrogen sorption also showed a very high specific surface area and significant pore volume, especially for the materials modified by grafting functional groups. On the other hand, for the materials modified by co-condensation, the values of textural parameters changed with the increasing number of the introduced functional groups: this was manifested, among others, by a significant increase in wall thickness at the expense of the total pore volume (Table 2). While in the case of the material containing the smallest number of functional groups introduced by co-condensation, the changes in textural parameters compared to the KIT-5/E reference sample are not so apparent, the changes in the mesoporous structure become much more pronounced in the case of the KIT-5/C/SO_3_H/0.100 sample. The exception is the D_me_ parameter, which in the case of the KIT-5/E material indicates larger cage sizes in this material than in the functionalized samples. However, it should be taken into consideration that the materials modified with co-condensation were obtained in separate synthesis processes, during which various disruptions in mesophase formation can occur, which in turn can result in non-homogeneous pore expansion [37]. Similar observations were also described by Yang and co-workers, who observed significant differences in the D_me_ parameter for the unmodified material and its analogs functionalized with propylamine groups introduced by co-condensation [44].

However, these changes upon structural and textural parameters during functionalization of the ordered mesoporous silica are a common feature, regardless of the type of applied sulfonic group precursor. Examples include studies conducted with 1,4-butanesultone, which was introduced into KIT-5 [25] and MCM-41 materials [47], and 3-((3-(trimethoxysilyl)propyl)thio)-propane-1-sulfonic acid used to functionalize MCM-41 silica [48]. Similar studies were reported by Mirsafaei et al. [49] and Vekariya et al. [50], where chlorosulfonic acid solution in 1,2-dichloromethane was used as a precursor of sulfonic groups to modify KIT-5 and MCM-41 samples. On the other hand, Tran et al. [51] modified another material from KIT family (KIT-6) using 3-mercaptopropyl(methyl)dimethoxysilane. In all papers mentioned above, the authors observed a decrease in the degree of structure ordering, which was visible on XRD patterns, and significant changes in textural parameters, namely specific surface area and pore size, when compared with the unmodified materials. It was despite the fact that in most cases the grafting procedure was used, which has no direct influence on the type of mesophase formed during silica matrix synthesis. In the case of co-condensation, this influence is usually even more pronounced [52]. Nevertheless, all materials synthesized during our study were still characterized by very good textural parameters, which indicates the occurrence of the ordered structure typical of KIT-5 materials [20], and confirms that the synthesis parameters were correctly selected.

TEM microscopy also confirmed the formation of ordered mesoporous structure in the synthesized samples. It revealed the presence of very well-structured nanopores in the obtained materials, which persists at each stage of functionalization, especially when it was performed by grafting [20,24]. The samples modified with the highest amount of –SO_3_H groups introduced by co-condensation possess a slightly less ordered structure, which was also proved by TEM analysis.

Elemental analysis allowed confirmation of the presence of sulphur atoms in the obtained materials and thus to positively verify the developed procedures of functionalization of nanostructural silica matrices. Comparing the two different modification methods, it can be seen that for the samples containing more MPTMS, the effectiveness of the introduction of functional groups is higher by co-condensation than by grafting. Furthermore, assessment of the elemental analysis results for the samples prior to oxidation and after this process, leading to –SH transformation to –SO_3_H, permitted evaluation of the effect of oxidation on the stability of binding of sulphur atoms in the synthesized materials.

The analysis of surface acidity proved that the introduction of sulfonic groups by the developed synthesis procedures leads to the generation of acidic active centers in KIT-5 silica materials. The acidity of the samples modified by co-condensation was much higher than that of those functionalized by grafting. Therefore, the density of the distribution of acidic centers per unit of specific surface area was also higher for the samples modified by co-condensation. Additionally, when comparing the results obtained from two different titration procedures, it can be observed that they are almost identical, which makes it possible to conclude that the acid-base titration can serve as a reliable method to determine the amount of active acidic centers [53]. Since the acid-base titration is not the best choice for the determination of –SH groups (e.g., see Ref. [54]), during our studies the amount of introduced functional groups before (–SH) and after the oxidation process (–SO_3_H) was determined by an elemental analysis. This made it possible to determine the amount of functional groups eluted during the oxidation step.

The particle size distributions for materials obtained by co-condensation or grafting with the same amount of functional groups are quite similar. Nevertheless, in the case of the grafting procedure the influence of the oxidation step on the particles sizes is more pronounced than for materials obtained by co-condensation, most likely due to the increased hydrophilicity of particles and, consequently, the reduced tendency to the formation of agglomerates. The results obtained by laser diffraction are also confirmed by SEM photographs showing irregular particles of non-uniform size with a smoothed surface, as well as agglomerates with bigger sizes. This is also in a good agreement with observations made, among others, by Chermahini et al. [25] and Mirsafaei et al. [49], where changes in the particle sizes of KIT-5 material have also been noticed upon its modification with sulfonic groups. It is worth mentioning, that by measuring the particle size of powder materials it is possible to examine the relationship between the particle size and bulk density, which may be important for their applications in catalytic systems. Moreover, these measurements can be used to evaluate the influence of particular synthesis steps on the particle sizes in the obtained catalysts.

Synthesized nanostructural materials modified with sulfonic groups, being active acidic centers, showed high catalytic activity in the Friedel–Crafts alkylation between anisole and benzyl alcohol. The main product in this reaction is p-benzylanisole. The second product, substituted in the ortho position (o-benzylanisole), is formed in smaller amounts due to the influence of the methoxyl substituent present in the anisole molecule. Dibenzyl ether is formed in the smallest amount. However, as the reaction proceeds, the amount of both p-benzylanisole and o-benzylanisole increases while the amount of dibenzyl ether decreases. Therefore, it can be concluded that part of dibenzyl ether decomposes and participates in the formation of the desired reaction products (Figure 5). Ramanathan et al. have observed that dibenzyl ether, formed by condensation of two molecules of benzyl alcohol, is the primary product that acts as an alkylating agent. They have also demonstrated the importance of the availability and form of acid sites not only in the conversion of benzyl alcohol but also in the transformation of dibenzyl ether [45].

The catalytic activity is strictly dependent on the availability of the active centers. This is particularly evident in the case of sample KIT-5/C/SO_3_H/0.100 for which, despite the large amount of the introduced functional groups, as shown by the results of elemental analysis (Table 3), the catalytic activity is not satisfactory. Although this sample has the fastest initial rate compared to other materials, the expected increase in benzyl alcohol conversion is not obtained over the progression of the reaction time. Therefore, it can be concluded that the introduced functional groups block part of the pore inlets and the reactant molecules cannot diffuse freely into the pores of the catalyst, resulting in a persistently low level of catalytic activity. Most likely, some of the functional groups during synthesis of the catalyst have located into the bulk of the material, thus becoming inaccessible to the reactants. Additionally, the changes in the structure that became particularly apparent in the D_me_ and V_tot_ values (Table 2), may have further strengthened the diffusional limitations to the active centers. Thus, it can be seen that in the case of lower loading with functional groups, modification by co-condensation helps to achieve a more uniform dispersion of the active centers than grafting. In the case of grafting, the effect of the availability of functional groups for the reactants also becomes apparent, especially when larger amounts of –SO_3_H groups are introduced: despite the nominally larger amount of functional groups, the expected increase in the catalytic activity is not obtained for samples KIT-5/G/SO_3_H/0.300 and KIT-5/G/SO_3_H/0.500.

Nevertheless, the KIT-5 based catalysts have a potential to be an alternative solution to homogeneous catalysts, e.g., AlCl_3_, BF_3_, H_2_SO_4_, HF, H_3_PO_4_, or p-toluenesulfonic acid. This was shown in our comparative study, in which the catalytic activity of the KIT-5/C/SO_3_H/0.100 catalyst was compared with results obtained in the presence of p-toluenesulfonic acid (PTSA). As it was shown in Table 5, the conversion of benzyl alcohol after 24 h of reaction for the homogeneous system is significantly lower than for the heterogeneous catalyst, reaching values of 3% and 42%, respectively. This seems rather surprising, but a similar trend was also observed by Melero and co-workers, who compared the activity of PTSA with SBA-15 bearing arenesulfonic groups in the Friedel–Crafts acylation of anisole with acetic anhydride. They also noted that the introduction of the sulfonic groups into the siliceous mesoporous structure leads to increased activity of the acid centers as compared with the homogeneous catalyst [55]. In addition, studies on a similar reaction (acylation of anisole with benzoic anhydride) by Koujout and Brown indicated a very significant effect of the reaction environment on the catalytic activities obtained, as the polarity of the solvent plays an important role in controlling the acid strength of the acid catalysts. Anisole, which exhibits low polarity and solvates sulfonic acid groups only very weakly, would lead to the negligible dissociation of the sulfonate anion, and the interactions between the anion and the undissociated acid to enhance acid strength would not occur. Therefore, even though the amount of sulfonic groups introduced by us to the homogeneous and heterogeneous catalytic system was analogous, the influence of the anisole weakened the acid strength of the PTSA catalyst [56]. This is also in good agreement with the results obtained by van Grieken et al. who proved that the arene-SO_3_H moieties show a more hydrophilic character than the propyl-SO_3_H ones. Hence in the catalytic systems with chemical species showing differences in polarity, the hydrophilic-hydrophobic balance of the catalyst cannot be neglected [57]. The correlations presented above help to explain why in the catalytic systems we have tested the heterogeneous catalyst KIT-5/C/SO_3_H/0.100, possessing propylsulfonic groups, proved to be much superior to the PTSA catalyst with a p-toluene substituent attached to the sulfonic group.

In addition, it is also worth noting the different distribution of products in the reaction we conducted (Table 5), which indicates a preference for the etherification reaction over the alkylation process when applying a homogeneous catalyst. This manifests itself in an increasing selectivity toward dibenzyl ether at the expense of alkylation products. Similar results were obtained by de la Cruz and co-workers, who also analyzed Friedel–Crafts alkylation involving anisole and benzyl alcohol in the presence of niobium phosphate as the catalyst [58]. Based on the studies mentioned above and those conducted by Rác et al. on the alkylation of benzene and toluene in the presence of benzyl alcohol, we can conclude that in the presence of a homogeneous catalyst, benzyl alcohol effectively competes with the aromatic reactants for the electrophile, namely acidic active centers, leading to the higher production of dibenzyl ether [33].

Consequently, our study shows that the KIT-5/C/SO_3_H/0.100 catalyst achieves better values of benzyl alcohol conversion and selectivity toward alkylation products than PTSA. This allowed us to confirm the thesis that catalysts prepared on the basis of KIT-5 mesoporous silica can be an important alternative to homogeneous systems in the Friedel–Crafts alkylation.

The KIT-5 materials synthesized in the present study exhibit high catalytic activity, which is slightly superior to that described for other mesoporous silica-based materials. For example, MCM-41 silica, containing an even higher number of functional groups than the KIT-5 samples presented here, allows one to obtain less than 40% conversion of benzyl alcohol after 4 h of reaction [9], while for KIT-5/G/SO_3_H/0.100 under analogous conditions this conversion after 4 h is as high as 80%.

An important aspect of catalytic research using mesoporous silicas as carriers of the active center is the influence of textural parameters on catalytic performance. Considering Friedel–Crafts reactions, only a few examples of alkylation reaction between anisole and benzyl alcohol can be found in the literature, which show the influence of the size and symmetry of mesopores on the profile of the obtained products. As mentioned already, studies by Kamegawa et al. [9] indicated that propylsulfone groups anchored on the surface of MCM-41 with 2D hexagonal pores yield lower conversion values for benzyl alcohol than is the case with the KIT-5/G/SO_3_H/0.100 catalyst prepared during our studies (~40% vs. ~80% after 4 h of reaction). This indicates the advantage of a silica matrix with a 3D pore network over materials based on a 2D channel network. Attempts by Kamegawa et al. to increase the catalytic activity by hydrophobizing the surface led to higher values of the turnover number (TON) calculated by the amount of benzylanisole formed per the number of SO_3_H moieties. However, the introduction of the hydrophobizing agent was accompanied by undesired changes in the mesoporous structure. An excess amount of the hydrophobic agent affected the catalytic performance by decreasing the accessibility of the reactants to the SO_3_H moieties on the surface of MCM-41 [9].

The influence of the support structure was also evident in the studies conducted by our group on acid catalysts prepared based on KIT-6 materials, which are characterized by a regular structure with Ia3d symmetry and a three-dimensional interpenetrating arrangement of cylindrical mesopores [59]. Sulfonic catalysts based on KIT-6 and KIT-5 behave similarly during the anisole alkylation reaction, namely, they promote the formation of the product substituted in the para position, that is, p-benzylanisole. In the case of both types of silicas, it can also be observed that as the reaction proceeds, the content of both isomers increases slightly, while the amount of dibenzyl ether decreases. However, in the case of catalysts based on KIT-6 materials, one can observe a higher conversion of benzyl alcohol obtained at the same time and a lower tendency to form an undesired product (dibenzyl ether). This may be due to a different way of connecting the pores into a three-dimensional network of channels that characterizes the KIT-6 materials, which is more conducive to the free flow of reactants than is the case with materials prepared based on KIT-5 silica, where smaller cage entrances can promote the formation of various types of blockages. In addition, our studies have shown that within a given mesoporous structure, changes in selectivity to particular products can also be induced by the application of microwave radiation, which in the case of our study led to a higher proportion of the desired reaction products in a shorter time of conducting the reaction, and furthermore to the formation of dibenzyl ether in smaller amounts than in the reaction carried out under conventional heating conditions [59]. Nevertheless, the 3D structure still promotes better selectivity in the Friedel–Crafts alkylation reaction compared to the 2D structure. Brown and co-workers also reached similar conclusions by studying the effect of the structure of mesoporous materials on the reaction of Friedel–Crafts alkylation of toluene with benzyl alcohol [18]. Studies have shown that for this reaction MCM-48 is a better support for sulfonic acid catalysts than MCM-41 and SBA-15, since, according to Brown et al., the accessibility of an active site plays a crucial role in controlling relative activities, and the 3D pore structure of MCM-48 results in better site accessibility than the structures in MCM-41 and SBA-15 [18].

On the other hand, studies by Ramanathan et al. showed that for the 3D structure of KIT-5 as a carrier of Lewis-type and to a small extent Brönsted-type active centers derived from Zr atoms, the selectivity to particular alkylation products can be modified by both the reaction temperature, the amount of catalyst used, and the molar ratio of the reactants [45]. Comparing the results of our study with those of Ramanathan et al., it can be seen that for the catalyst KIT-5/G/SO_3_H/0.100, after 6 h of reaction conducted at 100 °C, we obtained a conversion rate of benzyl alcohol of 92%, and the selectivity to the undesired product (dibenzyl ether) was only 16% (Table 5), while the results obtained by Ramanathan et al. after 6 h of reaction conducted at 90 °C were only 18% and more than 20% for conversion of benzyl alcohol and selectivity to dibenzyl ether, respectively. In contrast, raising the reaction temperature to 110 °C led to a sharp increase in benzyl alcohol conversion close to 100% and a reduction in selectivity to dibenzyl ether by almost half at the same reaction time [45]. Therefore, the results strongly suggest that alkylation activity depends on both the number and nature of the acid sites, and their accessibility is facilitated by the three-dimensional structure of the KIT-5 carrier.

Two different approaches to obtaining KIT-5 materials modified with –SO_3_H groups allowed us to evaluate how each functionalization method and the amount of functional groups introduced affect the quality of the obtained samples. Both co-condensation and grafting have their strengths and weaknesses [49], nevertheless, on the basis of our results it may be stated that in the case of Friedel–Crafts alkylation the materials obtained by grafting have better catalytic performance, because under analogous conditions they allow to obtain higher conversions of benzyl alcohol than their counterparts obtained by co-condensation (Figure 6). It was also observed that for both methods of functionalization there is a certain limit of functional groups, above which the influence of the amount of the modifying agent on catalytic activity disappears. It is particularly noticeable in the case of co-condensation, where the increase in the TEOS:MPTMS molar ratio from 0.050 to 0.100 no longer gives the expected increase in benzyl alcohol conversion. On the other hand, in the grafted materials introduction of a modifying agent, using amounts greater than 0.100—in the terms of the TEOS:MPTMS molar ratio—does not further increase the catalytic activity.

## 4. Materials and Methods

### 4.1. Reagents Used

The following chemical reagents were used in the procedures described below for the synthesis of KIT-5 materials: Pluronic F127 (ethylene oxide and propylene oxide triblock copolymer EO_106_PO_70_EO_106_, BASF, Ludwigshafen am Rhein, Germany), tetraethyl orthosilicate, TEOS (≥99%, Sigma-Aldrich, Darmstadt, Germany), 3-mercapropyltrimethoxysilane, MPTMS (95%, abcr GmbH, Karlsruhe, Germany), hydrochloric acid (37.3%, POCh, Gliwice, Poland), ethyl alcohol 96% (POCh, Gliwice, Poland), toluene (Chempur, Piekary Śląskie, Poland), and chloroform (98.5%, POCh, Gliwice, Poland). For the analysis of active acidic centres in the synthesized materials: sodium hydroxide (0.01 M, test portion, Chempur, Piekary Śląskie, Poland), sulphuric acid(VI) (0.005 M, test portion, Merck, Darmstadt, Germany) were used. In the Friedel–Crafts alkylation reaction the following materials were used: anisole (99%, Sigma-Aldrich, Darmstadt, Germany), benzyl alcohol (Ph Eur, Fluka Analytical, Darmstadt, Germany), n-decane (>94%, Merck, Darmstadt, Germany). Commercially available non-mesoporous silica (Sigma-Aldrich, Darmstadt, Germany) was used as a reference material in the heterogeneous catalytic tests, while p-toluenesulfonic acid, PTSA (≥98.5%, Sigma-Aldrich, Darmstadt, Germany) was used as a homogeneous catalyst.

### 4.2. Synthesis of Materials Modified with Sulfonic Groups by Grafting

#### 4.2.1. Synthesis of KIT-5

The synthesis of unmodified KIT-5 type materials was carried out on the basis of the procedure designed by Ryoo et al. [20]. It is briefly presented below: 5.0 g of Pluronic F127, 240 cm^3^ of distilled water, and 8.9 cm^3^ of HCl were introduced into PP bottle. The contents were stirred at 45 °C until the surfactant was completely dissolved. Then, 24 g of TEOS were added and stirring was continued for 24 h. The resulting suspension was placed in a laboratory oven (at 100 °C) for 24 h. After that, the preparation was filtered off and dried for 24 h at 100 °C. The molar ratios of individual reagents were: 1.00 TEOS: 3.5∙10^−3^ Pluronic F127: 0.88 HCl: 119 H_2_O. The next step in the synthesis of unmodified KIT-5 materials was to remove the template (Pluronic F127) from the pores of the obtained material. For this purpose, calcination (120 °C/2 h + 550 °C/2 h with 1.5 °C/min increment) was used, which was preceded by 30-min extraction with 0.1 M HCl solution in 96 % EtOH. The material obtained in this way (KIT-5/K) served as a carrier for acidic functional groups introduced by grafting. An additional sample was also prepared in which the template was removed exclusively by ultrasound-assisted extraction (0.1 M HCl in 96% EtOH, 60 °C, 6 h). This material (KIT-5/E) served as a reference for samples modified by co-condensation.

#### 4.2.2. Modification with MPTMS

MPTMS as a precursor of sulfonic groups was added in such quantities as to obtain different molar ratios of TEOS to MPTMS, which made it possible to determine the effect of added organosilane on the structural and textural parameters of the obtained materials as well as on their potential catalytic activity in the test reaction. The molar ratios of TEOS: MPTMS in the final samples are shown in Table 1. In order to graft the thiol groups on the surface of the calcined silica materials, 1 g of KIT-5 material was weighed into a glass vial and 98 cm^3^ of solvent (toluene) was added. An appropriate amount of MPTMS was introduced to the resulting mixture, depending on the molar ratio of TEOS and MPTMS, namely 0.015 cm^3^, 0.031 cm^3^, 0.081 cm^3^, 0.155 cm^3^, 0.309 cm^3^, 0.618 cm^3^, 0.929 cm^3^, 1.235 cm^3^ and 1.547 cm^3^ for TEOS:MPTMS ratios 1:0.005, 1:0.010, 1:0.025, 1:0.050, 1:0.100, 1:0.200, 1:0.300, 1:0.400, and 1:0.500, respectively. The grafting process was carried out in a nitrogen atmosphere for 24 h, at 90 °C. Then the functionalized silica material was washed (initially with toluene and then with chloroform) to remove unbound MPTMS and dried (80 °C/24 h).

#### 4.2.3. Oxidation of Thiol to Sulfonic Groups

Thanks to the application of MPTMS, the silica surface was enriched in thiol groups, however, obtaining catalytically active acid centres required oxidation of –SH to –SO_3_H groups. The oxidation procedure used by us consisted in adding 30 cm^3^ of H_2_O_2_ to the 1 g of sample containing thiol groups and stirring at room temperature for 24 h. After that, the preparation was washed, rinsed several times with distilled water and dried—first at room temperature and then in a vacuum dryer (35 °C/24 h).

### 4.3. Synthesis of Materials Modified with Sulfonic Groups by Co-Condensation

The synthesis was carried out in a PP bottle to which 1.25 g of Pluronic F127 surfactant, 60 cm^3^ of distilled water and 2.23 cm^3^ of HCl were introduced. The contents were stirred at 45 °C until the surfactant was dissolved, then 6 g of TEOS were added and stirred for another 30 min. After that the appropriate amount of MPTMS was added and the stirring was continued for 23.5 h so that the total hydrolysis and condensation time of TEOS was 24 h. The obtained sludge was placed in a laboratory dryer (100 °C/24 h), after which the resulting solid was filtered off and left to dry at room temperature. The next stage of the synthesis was the removal of the directing agent by extraction in the presence of ultrasounds. The extraction medium was 0.1 M HCl solution in 96% EtOH and the extraction process was carried out at 60 °C for 6 h. The samples were then washed several times with ethyl alcohol and allowed to dry at room temperature. The last step was to oxidize the introduced thiol groups to sulfonic ones. This reaction was carried out according to the scheme described above (Section 4.2.3). As a result of the above described grafting and co-condensation procedures, a series of KIT-5 materials with different functional group contents were obtained.

### 4.4. Characterization of Catalysts

X-ray diffraction studies in the low angle range of 2θ (0.6°–8.0°, CuKα, λ = 0.154 nm) were carried out with the use of Bruker AXS D8 Advance diffractometer (Bruker, Billerica, MA, USA), equipped with Johansson monochromator and LynxEye strip detector. The materials were analysed in beam knife-edge configuration, which reduced the background scattering occurring at low angles of 2θ. On the basis of the position of the main diffraction reflection, the distances between parallel network planes were determined, thus obtaining the values of parameter d_111_, used to calculate the distance between the centres of adjacent mesopores ($a_{0}=d_{111}*\sqrt{3}$). Measurements of low-temperature (−196 °C) nitrogen adsorption/desorption were performed on the Nova 1200e sorptometer (Quantachrome Instruments, Boynton Beach, FL, USA) in the range of relative pressures p/p_0_ from 0.02 to 1.00. The results of these measurements allowed the calculation of specific surface area (BET), average pore size, and their total volume (N_2_ at 77 K on the silica–cylindrical/spherical pore, NLDFT ads. model) [46]. Knowing the distances between the centres of the adjacent mesopores (a_0_) and the pore size calculated on the basis of XRD studies, it is also possible to calculate the wall thicknesses of the silica obtained. Due to the specific structure of KIT-5 type silica, in which the pores have narrow entrances and wide interiors (as already shown in Figure 1), additional parameters should be considered when calculating the wall thicknesses. On the basis of the equation developed by Ravikovitch and co-workers [60], the cage diameter is determined: $D_{me}=a_{0}*\sqrt[{3}]{\frac{6\epsilon_{me}}{\pi \nu }}$, where *ϵ_me_* is the volume fraction for a cage of cubic symmetry, while ν is the number of cages present in the unit cell (for the spatial group Fm3m, ν = 4). The value of *ϵ_me_* was found from the equation: $\epsilon_{me}=\frac{\rho_{\nu }V_{me}}{1+\rho_{\nu }V_{tot}}$ [60], where: *ρ*_V_ is the real wall density of a solid (for silica solids a value of ~2.2 g/cm^3^ is assumed), *V_me_* is the volume of mesopores [cm^3^/g] and *V_tot_* is the total volume of pores [cm^3^/g], which is determined from the equation: $V_{tot\;}=V_{me}+V_{mic}$. The value of *V_mic_*, i.e., the volume of internal pores (micropores and narrow mesopores) connecting large cages [cm^3^/g], was calculated using the alpha-s method [61]. After calculating all the parameters presented above, on the basis of the equation: $W=\frac{D_{me}}{3}*\frac{\left(1-\epsilon_{me}\right)}{\epsilon_{me}}$ [28,60], the average wall thickness of KIT-5 type materials was determined. Microscopic images of the materials were obtained using a transmission electron microscope JEM 1200 EX II (JEOL, Akishima, Tokyo, Japan) working with an electron beam with an acceleration voltage of 80 kV. Additionally, high-resolution Quanta 250 FEG (FEI, Hillsboro, OR, USA) scanning electron microscope was used. SEM images were taken in a low vacuum mode, with a chamber pressure of 70 Pa. Elemental analysis of the obtained materials was performed on the Elemental Analyser Vario EL III apparatus (Elementar Analysensysteme GmbH, Langenselbold, Germany). The measurement method consisted of catalytic combustion of the sample of exact weight (10–20 mg) at 1200 °C and analysis of the composition of gas combustion products on the basis of differences in their thermal conductivity. Thanks to these measurements, the percentages of carbon, hydrogen, and sulphur were determined, which permitted, among other things, the calculation of the so-called synthesis efficiency coefficient (R), i.e., the ratio of sulphur atoms introduced during the modification and determined using elemental analysis (S_AE_) to their assumed amount in the sample (S_theor._). Quantitative determination of acidic active centres in the obtained samples was carried out using the two direct and reverse techniques of acid-base titration. The principle of direct titration was to titrate a strictly defined mass of a given material with 0.01 M NaOH solution in the presence of phenolphthalein as the pH indicator. In the reverse titration an excess of 0.01 M NaOH solution was added to a specified mass of a given material and titration with 0.005 M H_2_SO_4_ solution in the presence of phenolphthalein as the pH indicator. For each procedure, the measurements for a given catalyst were repeated twice and the results obtained were averaged.

Particle size distributions for synthesized materials were measured by laser diffraction (LD) technique, using a Mastersizer 2000 device (Malvern Panalytical, Worcestershire, UK) equipped with a Hydro dispersion unit and a helium-neon laser generating light of λ = 632.8 nm and a blue semiconductor light source generating light of λ = 466 nm. Distilled water was used as the dispersion medium. The measurements were repeated five times and averaged for each sample to ensure repeatability.

### 4.5. Friedel–Crafts Alkylation Reaction

The Friedel–Crafts alkylation reaction was selected for the catalytic activity tests. The procedure was as follows: 0.050 g of catalyst, 5.435 cm^3^ of anisole, 0.515 cm^3^ of benzyl alcohol, and 0.500 cm^3^ of n-decane (internal standard) were placed in the reaction vessel. The reaction mixture was heated at 100 °C for 24 h, samples for analysis were collected after the 1st, 2nd, 3rd, 6th, and 24th hour from the beginning of the reaction. The reaction products were identified by means of chromatographic techniques: initially with a gas chromatograph coupled to a VARIAN CP-3800 mass detector (Varian, Inc., Palo Alto, CA, USA), equipped with an automatic injector, an ion trap mass analyser, and a VF-5MS capillary column of 30 m in length, 0.25 mm internal diameter, and 0.25 μm film thickness, followed by a Bruker 430-GC gas chromatograph (Bruker, Billerica, MA, USA) equipped with an automatic injector, a flame ionisation detector (FID), and a DB-5MS capillary column 30 m long, 0.25 mm inside diameter, and 0.25 μm film thickness.

In the case of catalytic tests with a homogeneous catalyst, namely p-toluenesulfonic acid (PTSA), the procedure was analogous to the one described for heterogeneous catalysis, but with a different amount of catalyst applied. The quantity of PTSA was 7.4 mg, which made it possible to introduce an amount of active sites corresponding to those of heterogeneous systems with KIT-5/C/SO_3_H/0.100 catalysts. The reaction mixture was heated at 100 °C for 24 h, and samples were collected and analysed in the same intervals as for tests involving heterogeneous catalysts.

## Figures and Tables

**Table 1:** Unit cell parameters of KIT-5 materials modified with different molar ratios of TEOS: MPTMS introduced by grafting or co-condensation.

Sample	Functionalization Procedure	Molar Ratio TEOS:MPTMS	2θ [°]	d_111_ [nm]	a_0_ [nm]
KIT-5/K	---	---	0.84	10.5	18.3
KIT-5/G/SO_3_H/0.005	grafting	1:0.005	0.82	10.8	18.8
KIT-5/G/SO_3_H/0.010	grafting	1:0.010	0.86	10.3	17.9
KIT-5/G/SO_3_H/0.025	grafting	1:0.025	0.83	10.3	17.9
KIT-5/G/SO_3_H/0.050	grafting	1:0.050	0.81	10.8	18.8
KIT-5/G/SO_3_H/0.100	grafting	1:0.100	0.84	10.6	18.3
KIT-5/G/SO_3_H/0.200	grafting	1:0.200	0.84	10.6	18.3
KIT-5/G/SO_3_H/0.300	grafting	1:0.300	0.88	10.1	17.5
KIT-5/G/SO_3_H/0.400	grafting	1:0.400	0.86	10.3	17.9
KIT-5/G/SO_3_H/0.500	grafting	1:0.500	0.90	9.9	17.1
KIT-5/E	---	---	0.74	12.0	20.8
KIT-5/C/SO_3_H/0.005	co-condensation	1:0.005	0.98	9.1	15.7
KIT-5/C/SO_3_H/0.010	co-condensation	1:0.010	0.86	10.3	17.9
KIT-5/C/SO_3_H/0.025	co-condensation	1:0.025	0.82	10.8	18.8
KIT-5/C/SO_3_H/0.050	co-condensation	1:0.050	0.86	10.3	17.9
KIT-5/C/SO_3_H/0.100	co-condensation	1:0.100	0.95	9.3	---

2θ—position of main reflection; d_111_—distances between parallel network planes (111); a_0_—unit cell parameter.

**Table 2:** Textural parameters of KIT-5 type materials modified with sulfonic groups introduced by grafting or co-condensation.

Sample	S_BET_	D_DFT_	V_tot_	V_mic_	D_me_	W
	[m^2^/g]	[nm]	[cm^3^/g]	[cm^3^/g]	[nm]	[nm]
KIT-5/K	669	2.1	0.39	0.16	9.3	8.1
KIT-5/G/SO_3_H/0.005	578	2.1	0.35	0.08	10.3	6.5
KIT-5/G/SO_3_H/0.010	566	2.1	0.35	0.09	9.6	6.7
KIT-5/G/SO_3_H/0.025	545	2.1	0.35	0.09	9.6	6.6
KIT-5/G/SO_3_H/0.050	584	2.1	0.35	0.09	10.1	6.9
KIT-5/G/SO_3_H/0.100	453	2.1	0.29	0.05	9.8	7.0
KIT-5/G/SO_3_H/0.200	478	2.2	0.30	0.08	9.4	7.9
KIT-5/G/SO_3_H/0.300	480	2.0	0.30	0.08	9.1	7.2
KIT-5/G/SO_3_H/0.400	457	2.0	0.28	0.09	9.0	8.2
KIT-5/G/SO_3_H/0.500	449	2.1	0.28	0.07	8.8	7.4
KIT-5/E	398	2.6	0.30	0.04	11.3	7.3
KIT-5/C/SO_3_H/0.005	402	2.6	0.29	0.04	8.5	5.8
KIT-5/C/SO_3_H/0.010	312	2.6	0.22	0.04	9.0	8.1
KIT-5/C/SO_3_H/0.025	243	2.6	0.17	0.03	9.0	9.9
KIT-5/C/SO_3_H/0.050	288	2.0	0.19	0.05	8.4	10.0
KIT-5/C/SO_3_H/0.100	308	2.0	0.17	0.05	7.3	10.1

S_BET_—surface area (BET); D_DFT_—pore diameter (DFT); V_tot_—total pore volume (DFT); V_mic_—micropore volume (alpha-s); D_me_—cage size; W—wall thickness (DFT and XRD).

**Table 3:** Results of elemental analysis for KIT-5 materials modified with thiol or sulfonic groups by grafting or co-condensation.

Sample	%C	%H	%S		R
			S_AE_	S_theor._	
KIT-5/G/SH/0.005	0.93	0.74	0.25	0.25	1.00
KIT-5/G/SO_3_H/0.005	0.75	1.56	0.24		0.97
KIT-5/G/SH/0.100	3.32	1.00	1.70	3.87	0.44
KIT-5/G/SO_3_H/0.100	1.58	1.63	0.85		0.22
KIT-5/G/SH/0.500	3.97	1.67	2.61	9.94	0.26
KIT-5/G/SO_3_H/0.500	1.80	1.97	1.30		0.13
KIT-5/C/SH/0.005	13.00	2.95	0.23	0.25	0.92
KIT-5/C/SO_3_H/0.005	9.52	2.83	0.22		0.88
KIT-5/C/SH/0.100	13.82	4.02	3.12	5.06	0.62
KIT-5/C/SO_3_H/0.100	9.15	2.60	2.74		0.54

S_AE_—% content of sulphur obtained from elemental analysis; S_theor._—% content of sulphur assumed to be introduced; R = S_AE_/S_theor._ (synthesis effectiveness coefficient).

**Table 4:** Acidity analysis of KIT-5 type materials modified with sulfonic groups by grafting or co-condensation.

Sample	Acidity [mmol H^+^/g]		Acid Centres Density [–SO_3_H/nm^2^]
	Direct Titration	Reverse Titration	
KIT-5/K	0.04	---	---
KIT-5/G/SO_3_H/0.005	0.05	0.05	0.05
KIT-5/G/SO_3_H/0.010	0.05	---	0.05
KIT-5/G/SO_3_H/0.025	0.06	---	0.07
KIT-5/G/SO_3_H/0.050	0.07	---	0.07
KIT-5/G/SO_3_H/0.100	0.12	0.11	0.16
KIT-5/G/SO_3_H/0.200	0.15	---	0.19
KIT-5/G/SO_3_H/0.300	0.15	0.15	0.19
KIT-5/G/SO_3_H/0.400	0.17	---	0.22
KIT-5/G/SO_3_H/0.500	0.19	0.19	0.25
KIT-5/E	0.04	---	---
KIT-5/C/SO_3_H/0.005	0.10	0.10	0.15
KIT-5/C/SO_3_H/0.010	0.12	---	0.23
KIT-5/C/SO_3_H/0.025	0.13	---	0.32
KIT-5/C/SO_3_H/0.050	0.16	0.17	0.33
KIT-5/C/SO_3_H/0.100	0.26	0.27	0.51

KIT-5/K—non-modified sample after the removal of surfactant by calcination; KIT-5/E—non-modified sample after the removal of surfactant by extraction.

**Table 5:** Benzyl alcohol conversion and selectivity values for the products of Friedel–Crafts alkylation reaction carried out in the presence of KIT-5 type materials modified by sulfonic groups by grafting or co-condensation.

Sample	Reaction Time [h]	Selectivity [%]			Benzyl Alcohol Conversion [%]
		o-Benzylanisole	p-Benzylanisole	Dibenzyl Ether	
SiO2/G/SO_3_H/0.050	1	32	38	30	2
	6	32	39	29	6
	24	32	40	28	10
KIT-5/G/SO_3_H/0.050	1	33	41	26	7
	6	33	42	25	33
	24	33	43	24	61
KIT-5/G/SO_3_H/0.100	1	35	42	23	36
	6	38	46	16	92
	24	40	48	12	99
KIT-5/G/SO_3_H/0.500	1	37	42	21	59
	6	39	44	17	76
	24	39	44	17	82
KIT-5/C/SO_3_H/0.050	1	30	41	29	11
	6	33	43	24	64
	24	35	45	20	85
KIT-5/C/SO_3_H/0.100	1	33	41	26	25
	6	33	42	25	41
	24	33	42	25	42
PTSA	1	24	26	49	<1
	6	21	25	54	1
	24	15	23	62	3

## Data Availability

The data presented in this study are available upon request from the authors.

## Supplementary Materials

The following supporting information can be downloaded at: https://www.mdpi.com/article/10.3390/ijms24032165/s1.
