# Supplementary material for: KIT-5 Structural and Textural Changes in Response to Different Methods of Functionalization with Sulfonic Groups
Source: Int J Mol Sci. 2023 Jan 21;24(3):2165. doi: 10.3390/ijms24032165 (PMC9917139; doi:10.3390/ijms24032165)
Supplement: Supplementary file 1 [file ijms-24-02165-s001.zip › ijms-2109545-supplementary.pdf]

Supporting Information

KIT-5 Structural and Textural Changes in Response to Different Methods of Functionalization with Sulfonic Groups

Sylwia Chałupniczak, Izabela Nowak* and Agata Wawrzyńczak

Faculty of Chemistry, Adam Mickiewicz University in Poznań, Uniwersytetu Poznańskiego 8, 61-614 Poznań, Poland
* Correspondence: nowakizak@amu.edu.pl

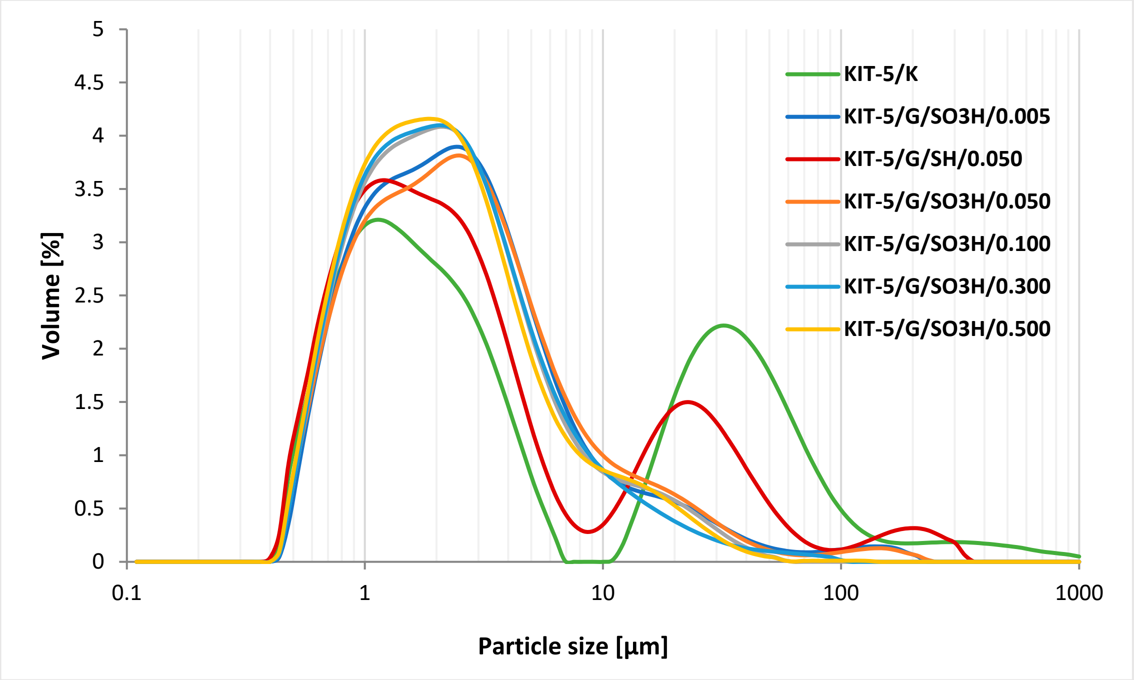

(**a**)

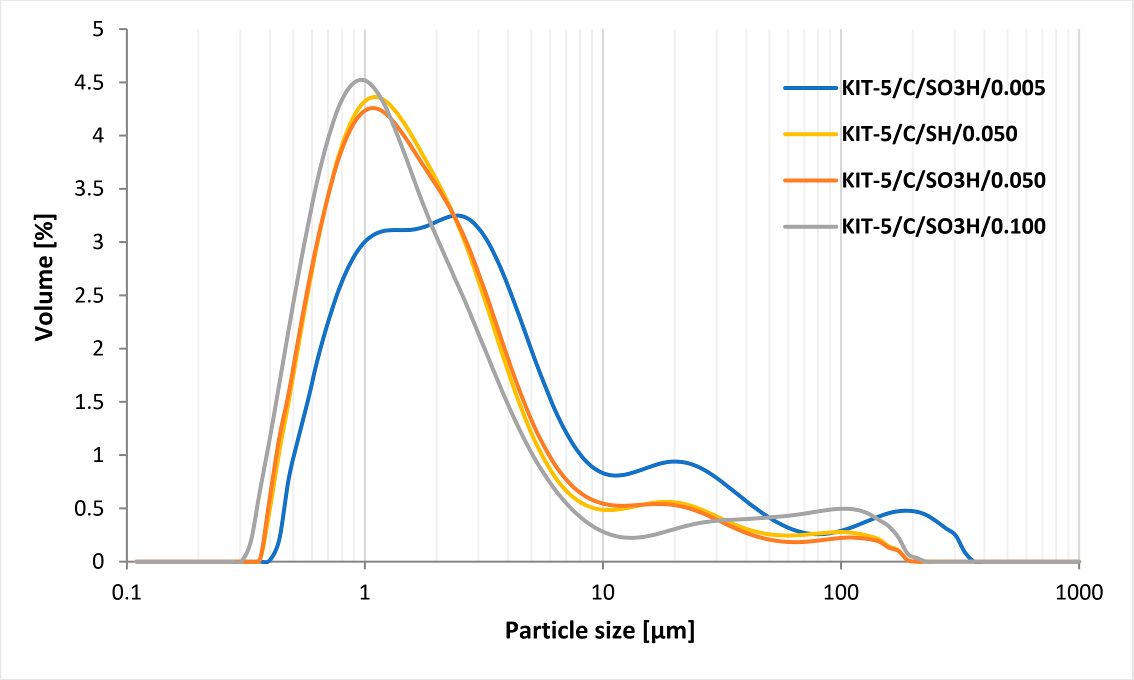

(**b**)

Figure S1. Comparison of the particle size distribution for the KIT-5 samples functionalized by (**a**) grafting or (**b**) co-condensation.

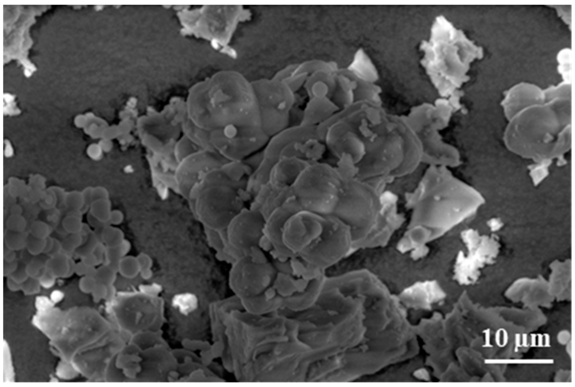

(**a**)

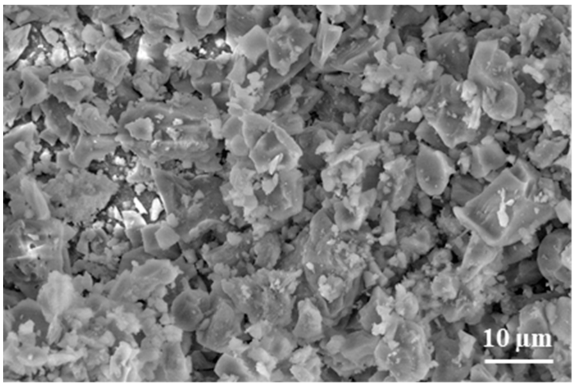

(**b**)

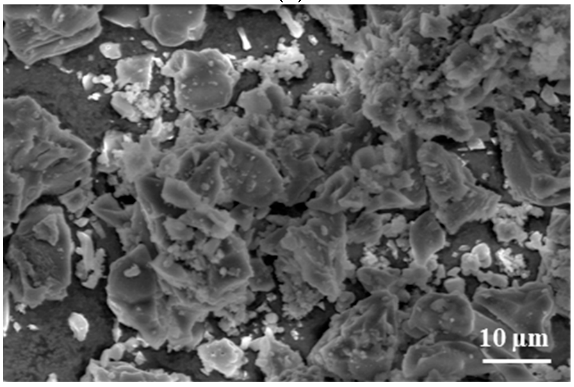

(**c**)

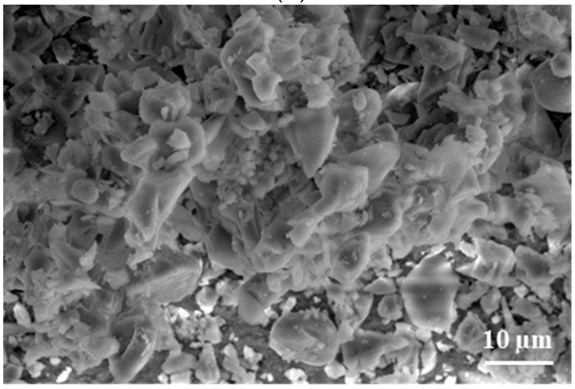

(**d**)

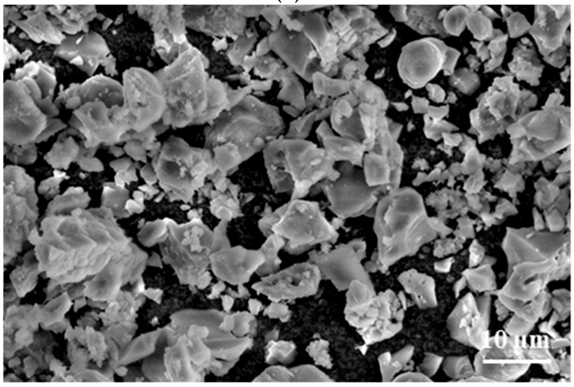

(**e**)

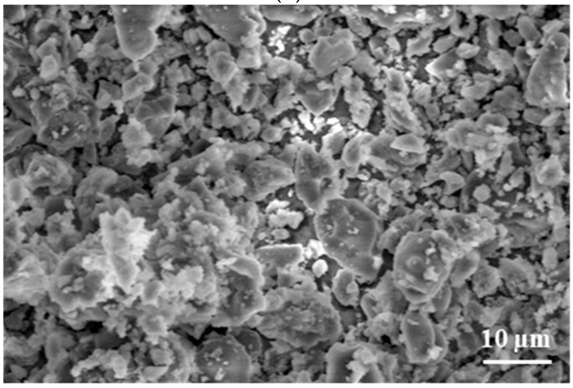

(**f**)

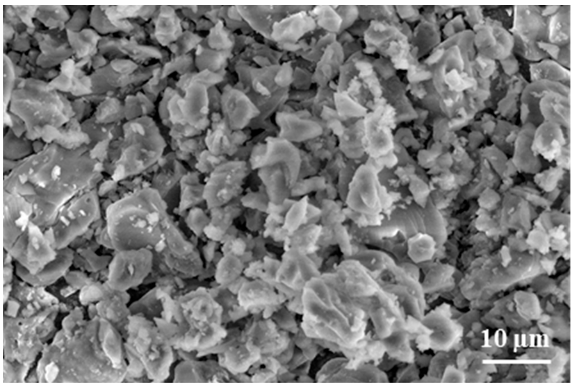

(**g**)

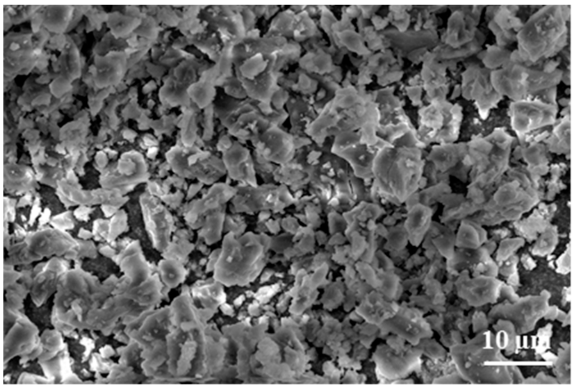

(**h**)

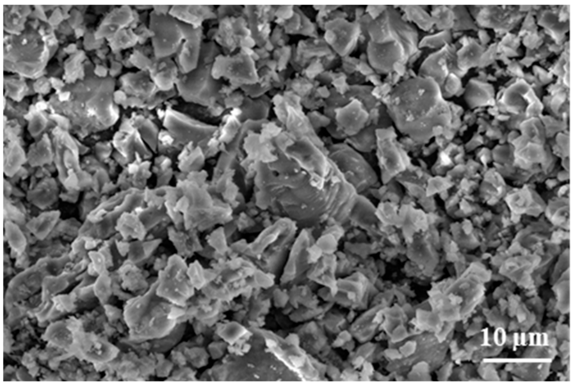

(**i**)

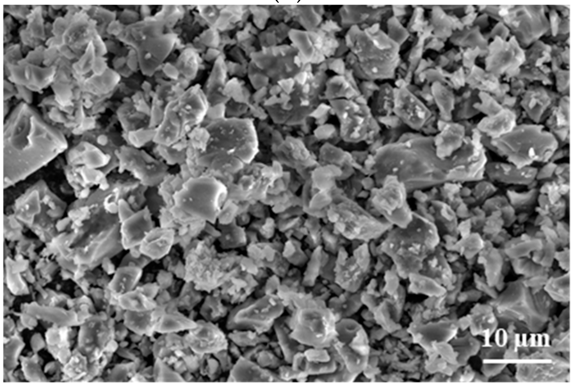

(**j**)

Figure S2. SEM micrographs of unmodified materials (**a**) KIT-5/K and (**f**) KIT-5/E, materials modified with sulfonic groups introduced by grafting: (**b**) KIT-5/G/SO3H/0.005, (**c**) KIT-5/G/SH/0.050, (**d**) KIT-5/G/SO3H/0.050, (**e**) KIT-5/G/SO3H/0.100 and by co-condensation: (**g**) KIT-5/C/SO3H/0.005, (**h**) KIT-5/C/SH/0.050, (**i**) KIT-5/C/SO3H/0.050, (**j**) KIT-5/C/SO3H/0.100.
